# Clinical efficacy of clopidogrel and ticagrelor in patients undergoing off-pump coronary artery bypass grafting: a retrospective cohort study

**DOI:** 10.1097/JS9.0000000000001246

**Published:** 2024-03-04

**Authors:** Zi Wang, Runhua Ma, Xiaoyu Li, Xiaoye Li, Qing Xu, Yao Yao, Chunsheng Wang, Qianzhou Lv

**Affiliations:** a Department of Pharmacy, Zhongshan Hospital, Fudan University; b Department of Cardiac Surgery, Zhongshan Hospital, Fudan University, Shanghai, People’s Republic of China

**Keywords:** bleeding, clopidogrel, major adverse cardiovascular event, off-pump coronary artery bypass grafting, ticagrelor

## Abstract

**Background:**

Ticagrelor is reportedly more effective than clopidogrel in preventing atherothrombotic events in patients with percutaneous coronary intervention. However, the optimal antiplatelet therapy strategy after off-pump coronary artery bypass grafting (OPCABG) is yet to be established.

**Materials and Methods:**

This study was performed using the prospectively-maintained database at our institution. Patients who underwent OPCABG were divided into the clopidogrel and the ticagrelor groups. Propensity score matching analysis was performed between the two groups. The clinical outcome was the occurrence of major adverse cardiovascular event (MACE), defined as a composite of vascular death, myocardial infarction, or stroke 1-year after surgery.

**Results:**

In total, 545 patients completed the entire follow-up assessment. After propensity score matching, 232 patients each were included in the clopidogrel and ticagrelor groups. The primary outcome occurred in 7.8 and 4.3% of patients in the clopidogrel and ticagrelor groups, respectively (*P*=0.113). *CYP2C19* variants (*2, *3, and *17) did not impact the clinical outcomes, regardless of the use of clopidogrel or ticagrelor. The rates of MACE were significantly lower in patients carrying the *ABCB1* C3435T CT/TT genotypes in the ticagrelor group than in those carrying the *ABCB1* C3435T CC genotype in the clopidogrel group (1.4 *vs.* 9.1%, adjusted *P*=0.030), as well as those carrying the *ABCB1* C3435T CC genotype in the ticagrelor group (1.4 *vs.* 8.9%, adjusted *P*=0.036). The *ABCB1* C3435T CC genotype was significantly associated with the incidence of 1-year MACE (HR=1.558, 95% CI: 1.109–2.188, *P*=0.011). Patients who experienced severe perioperative bleeding exhibited a significantly higher incidence of MACE than those who did not experience severe perioperative bleeding (14.0 *vs.* 4.9%, adjusted *P*=0.007).

**Conclusion:**

There was no significant difference in the 1-year MACE between patients receiving clopidogrel and those receiving ticagrelor after OPCABG. Notably, The *ABCB1* C3435T CC genotype was related to a higher risk of MACE.

## Introduction

Highlights

- No significant difference was found in 1-year major adverse cardiac event between patients receiving clopidogrel and those receiving ticagrelor after OPCABG.
- Individuals with *ABCB1* C3435T CC genotype had a significant association with the incidence of major adverse cardiovascular event at 1-year after OPCABG.
- The incidence of major adverse cardiovascular event was related to severe perioperative bleeding in patients undergoing OPCABG.

Coronary artery bypass grafting (CABG) remains an effective revascularisation procedure for patients with coronary artery disease. Importantly, CABG confers a survival benefit over medical therapy or percutaneous coronary intervention (PCI) among patients with left main or complex multivessel disease, diabetes, and reduced left ventricular function^1,2^. Despite this improvement, the occurrence of major adverse cardiovascular events (MACEs) following CABG poses a considerable challenge. More than 10% of patients reportedly experience adverse cardiovascular events in the first year post-CABG^3,4^. The conventional CABG procedure, which involves the use of cardiopulmonary bypass, also known as ‘on-pump’ CABG, is widely recognized as the benchmark procedure. To reduce the detrimental effects associated with cardiopulmonary bypass and the manipulation of the ascending aorta, off-pump coronary artery bypass grafting (OPCABG), which is performed on the beating heart with the aid of stabilising devices, was developed^5,6^.

To reduce the risk of thrombotic events in patients with acute coronary syndrome (ACS), the use of dual antiplatelet therapy (DAPT) with aspirin is recommended, combined with a P2Y_12_ receptor antagonist for 12 months, as secondary prevention after OPCABG^7,8^. Currently, clopidogrel and ticagrelor are the most commonly used P2Y_12_ receptor antagonists in clinical practice. Clopidogrel, a derivative of thienopyridine, undergoes conversion to its active metabolite through hepatic enzymes, specifically cytochrome P450 3A4 (CYP3A4) enzymes. The response to clopidogrel may be influenced by pharmacokinetic variables, such as intestinal absorption and metabolic activation in the liver, both of which can be impacted by genetic polymorphisms^9^. Ticagrelor is a reversible and direct-acting antagonist that exhibits a rapid onset and offset of the antiplatelet effect. Ticagrelor has a distinct chemical structure when compared with thienopyridines and does not warrant hepatic activation^10^. Randomised clinical trials have documented the superiority of ticagrelor over clopidogrel in preventing atherothrombotic events in patients with ACS undergoing PCI^11,12^. However, the optimal DAPT strategy for patients who have undergone CABG is yet to be established. Recently, MACEs were found to be similar between dual antiplatelet therapy with ticagrelor and dual antiplatelet therapy with clopidogrel post-CABG^13–15^. Conversely, a meta-analysis found that ticagrelor-based regimens could reduce mortality and MACEs without excess bleeding risk when compared with aspirin monotherapy or aspirin combined with clopidogrel in patients undergoing CABG^16^. Current guidelines recommend the use of clopidogrel or ticagrelor in patients with CABG; however, it remains unclear whether aspirin combined with clopidogrel or aspirin combined with ticagrelor should be the preferred antiplatelet therapy strategy after OPCABG surgery^7,17^.

In the current study, we aimed to compare the 1-year clinical outcomes between patients who received aspirin plus clopidogrel and those who received aspirin plus ticagrelor after OPCABG. Additionally, we aimed to determine the impact of genetic polymorphisms on the response to antiplatelet therapy in secondary prevention after cardiac surgery.

## Methods

### Study design and patients

This study was conducted using the prospectively-maintained database in our institution. The study was performed in accordance with the principles of the Declaration of Helsinki, and the protocol was approved by our regional ethics committee. All participants signed a written informed consent. This work has been reported in line with the Strengthening The Reporting Of Cohort Studies in Surgery (STROCSS) criteria^18^ (Supplemental Digital Content 1, http://links.lww.com/JS9/C57). We retrospectively reviewed the clinical data of consecutive patients who underwent OPCABG at our center between May 2020 and November 2021. Patients who met the following criteria were included in the current study: (1) aged ≥18 years; (2) underwent OPCABG; and (3) receiving dual antiplatelet therapy comprising either aspirin plus clopidogrel or aspirin plus ticagrelor after cardiac surgery. Patients were excluded if they: (1) had undergone other combined surgeries; (2) had any concomitant or previous malignancies; (3) changed their antiplatelet regimen during follow-up; (4) had incomplete clinical data; (5) were lost to follow-up; (6) received other antiplatelet agents during follow-up; or (7) received any anticoagulant agents during follow-up.

### Clinical management

All surgical procedures were performed using a median sternotomy. Systemic heparinization (1 mg/kg) was used, along with additional doses, to achieve an activated clotting time of more than 300 s. Once the surgery was finished, the effects of heparin were neutralized with protamine sulfate in a 1:1 ratio. A cell salvage device was used throughout the operation, with the salvaged blood being reinfused into the patient before the completion of the procedure. Following the surgery, all patients were transferred to the ICU.

The perioperative antiplatelet treatment was administered in accordance with the current guidelines and clinical practices at our center. Briefly, aspirin was typically continued at a low daily dose (100 mg) throughout the perioperative period. Ticagrelor was discontinued for a minimum of 3 days, and clopidogrel was discontinued for a minimum of 5 days prior to surgery. However, some patients underwent surgery prior to the scheduled date in cases deemed urgent. The cardiology team made the final decision, considering the patients’ symptoms and hemodynamic status, as well as estimating the risks of bleeding and ischemia. Aspirin and P2Y_12_ receptor antagonists (clopidogrel or ticagrelor) were typically resumed within 24 h postsurgery.

The actual antiplatelet treatment administered to patients at our center postsurgery depended on the surgeons’ advice and the patient’s compliance. According to the antiplatelet treatment postsurgery, the patients were divided into two groups: the clopidogrel group (patients receiving aspirin 100 mg daily plus clopidogrel 75 mg daily postsurgery) and the ticagrelor group (patients receiving aspirin 100 mg daily plus ticagrelor 90 mg twice daily postsurgery).

### Outcomes

The clinical outcome was the occurrence of MACE, defined as a composite of vascular death, myocardial infarction, or stroke during the 12 months postsurgery. Death from vascular causes was defined as death resulting from cardiovascular or cerebrovascular causes or any death without an identified cause. Myocardial infarction was defined based on the universal definition proposed in the Fourth Universal Definition of Myocardial Infarction in 2018^19^. Stroke was defined as a loss of neurological function in a specific area caused by either an ischemic or hemorrhagic event, resulting in residual symptoms lasting at least 24 h or death. The stroke diagnosis was confirmed by a neurology or neurosurgical specialist based on at least one brain imaging procedure, such as computed tomography, MRI, or cerebral vessel angiography^20^.

To investigate the relationship between perioperative bleeding and clinical outcomes, the Universal Definition of Perioperative Bleeding (UDPB) was used^21^. Universal Definition of Perioperative Bleeding-defined class 3 or class 4 bleeding was regarded as severe perioperative bleeding in this study^22^. Therefore, severe perioperative bleeding is defined by meeting one or more of the following six criteria: (1) chest tube drainage volume exceeding 1000 ml within the first 12 h after surgery; (2) delayed sternal closure; (3) surgical re-exploration due to bleeding; (4) use of recombinant factor VIIa; (5) transfusion of five or more units of red blood cells within 24 h of chest closure; (6) transfusion of five or more units of plasma within 24 h of chest closure.

Patients were followed up starting from the day of the operation. Routine tests for patients postsurgery included a physical examination, laboratory testing, ECG, and echocardiogram at our center. Follow-up assessments were performed during the 1st, 3rd, 6th, 9th and 12th months. The final follow-up date was performed in November 2022. Additionally, a routine telephone follow-up would be arranged if the patients were unable to attend the outpatient clinic.

### Genotyping

Two milliliters of peripheral whole blood samples were obtained from each patient in anticoagulated tubes containing ethylenediaminetetraacetic acid (EDTA) and stored at −80°C. Genomic DNA was extracted using a QIAamp DNA Mini Blood Kit (QIAGEN, Germany). Single nucleotide polymorphisms (SNPs) were genotyped using the Sequenom MassArray system and MassArray Typer 4.0 software (Sequenom)^23^. Two genes, *CYP2C19* and *ABCB1*, which are associated with responsiveness to antiplatelet therapy, particularly clopidogrel, were chosen for genotyping. The *CYP2C19* gene was analyzed for the CYP2C19*2, CYP2C19*3, and CYP2C19*17 polymorphisms, while the *ABCB1* gene was analyzed for the C3435T polymorphism.

### Statistical analysis

The Shapiro–Wilk test was used to assess the normality of variables. The data are presented as the mean±SD for continuous variables that follow a normal distribution, or as the median with interquartile range (25–75%) for variables that do not follow a normal distribution. Categorical variables are presented as numbers and percentages. Continuous variables between groups were compared using either Student’s *t*-test or the Mann–Whitney *U* test. Categorical variables were compared using the *χ*^2^ test or Fisher’s exact test if necessary. Comparisons among multiple groups were made using a one-way analysis of variance (ANOVA) with Bonferroni-corrected post-hoc comparisons. The Kruskal–Wallis test was used for non-normally distributed data. SNPs were tested for deviation from the Hardy–Weinberg equilibrium. Genotype frequencies were compared with the SNP database for the East Asian population.

In order to account for differences between groups, the propensity scores (PS) were developed to quantify the probability of each patient receiving either clopidogrel or ticagrelor. This was achieved by conducting a multivariable logistic regression analysis, taking into consideration the patient characteristics and clinical risk factors. These factors included age (>65 years), sex, BMI, hypertension, diabetes mellitus, hyperlipidemia, stroke, acute coronary syndrome, PCI, number of grafts, use of statin, use of β blocker, use of proton pump inhibitor, hemoglobin level, red blood cell level, activated partial thromboplastin time, and prothrombin time. Propensity score matching was performed using a 1:1 matching protocol with a caliper width equal to 0.1 of the SD of the logit of the PS.

Kaplan–Meier estimates were used to calculate and plot the cumulative risk of the first occurrence of outcome events, and the differences between groups were compared by the log-rank test. Cox regression was used to compare outcomes between treatment groups and between genotype groups in each treatment group, as well as analyze the interaction between treatment groups and genotype groups, with adjustment for covariates: age >65 years, sex, hypertension, diabetes mellitus, acute coronary syndrome, and use of proton pump inhibitor. In order to determine the factors related to MACE and perioperative bleeding, univariable, and multvariable analyses were performed. Patient baseline characteristics, patient comorbidities, antiplatelet therapy, surgical techniques, and other combined medications were included in the univariable analysis. Variables with *P*-value <0.1 in the univariable analysis were included in the multivariable logistic regression analysis.

All statistical analyses were conducted using SPSS 25.0 software and GraphPad Prism 8.0.2 statistical software. All results were considered statistically significant at *P*<0.05 (two-tailed).

## Results

### Patient characteristics

**Figure 1:**
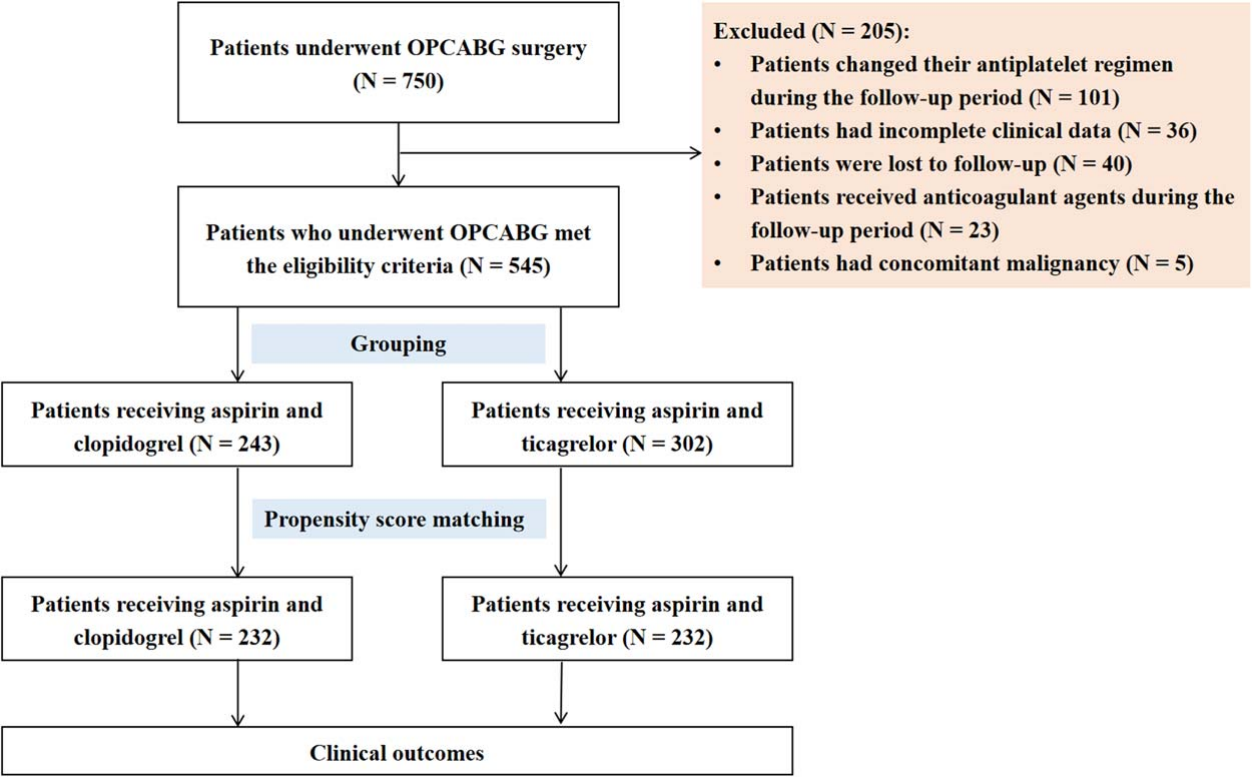
Flowchart.

During the study period, 750 patients underwent OPCABG surgery. Among these patients, 101 changed their antiplatelet regimen, 36 had incomplete clinical data, 40 were lost to follow-up, 23 received anticoagulant agents during the follow-up period, and 5 presented with a concomitant malignancy (Fig. 1). Finally, 545 patients who had undergone OPCABG met the eligibility criteria and completed the entire follow-up assessment. The clopidogrel and ticagrelor groups comprised 243 and 302 patients, respectively. Table 1 summarizes the baseline characteristics of patients. The proportions of patients in the ticagrelor group with hyperlipidemia, ACS, and those using proton pump inhibitors postsurgery were higher than those in the clopidogrel group. In contrast, the proportion of patients in the ticagrelor group who were over 65 years of age was lower than that in the clopidogrel group. Patients in the ticagrelor group had a higher BMI and red blood cell counts than patients in the clopidogrel group, with lower prothrombin levels. After propensity score matching, 232 patients each were included in the clopidogrel and ticagrelor groups, respectively. There were no significant differences in patient characteristics between the two groups after propensity score matching. Genotype distributions did not deviate from the Hardy–Weinberg equilibrium. The distribution of genotypes was compared with the SNP database (https://www.ncbi.nlm.nih.gov/snp/) and matched those reported for the East Asian population (Table 2).

**Table 1:** Baseline characteristics before and after matching.

	Before matching			After matching		
Variables	Clopidogrel (*N*=243)	Ticagrelor (*N*=302)	*P*	Clopidogrel (*N*=232)	Ticagrelor (*N*=232)	*P*
Age >65 years, *n* (%)	159 (65.4)	154 (51.0)	0.001	148 (63.8)	133 (57.3)	0.183
Male, *n* (%)	196 (80.7)	240 (79.5)	0.748	186 (80.2)	174 (75.0)	0.221
BMI, kg/m^2^	24.36±2.91	24.92±3.22	0.035	24.36±2.93	24.57±3.23	0.479
Hypertension, *n* (%)	177 (72.8)	211 (69.9)	0.505	168 (72.4)	162 (69.8)	0.609
DM, *n* (%)	116 (47.7)	142 (47.0)	0.931	111 (47.8)	114 (49.1)	0.853
Hyperlipidemia, *n* (%)	77 (31.7)	126 (41.7)	0.016	75 (32.3)	86 (37.1)	0.329
Stroke history, *n* (%)	32 (13.2)	40 (13.2)	1.000	31 (13.4)	27 (11.6)	0.674
ACS, *n* (%)	96 (39.5)	164 (54.3)	0.001	92 (39.7)	113 (48.7)	0.061
PCI history, *n* (%)	40 (16.5)	44 (14.6)	0.553	36 (15.5)	38 (16.4)	0.899
Number of grafts, *n*	2.92±0.78	3.03±0.87	0.143	2.93±0.79	2.96±0.85	0.692
HB, g/l	131.37±15.77	133.51±14.79	0.103	131.35±15.97	130.13±13.74	0.374
RBC, 10×12/l	4.30±0.55	4.41±0.51	0.011	4.31±0.54	4.31±0.46	0.931
APTT, s	27.11±2.42	27.12±2.28	0.957	27.12±2.44	27.00±2.37	0.606
PT, s	11.83±0.91	11.67±0.84	0.031	11.82±0.90	11.74±0.87	0.338
PPI, *n* (%)	207 (85.2)	282 (93.4)	0.003	205 (88.4)	214 (92.2)	0.209
Statin use, *n* (%)	228 (93.8)	289 (95.7)	0.337	217 (93.5)	221 (95.3)	0.546

ACS, acute coronary syndrome; APTT, activated partial thromboplastin time; DM, diabetes mellitus; HB, hemoglobin; PCI, percutaneous coronary intervention; PPI, proton pump inhibitor; PT, prothrombin time; RBC, red blood cell.

**Table 2:** Distribution of variant genotypes within the study population.

Rs number	Number, *n* (%)	Minor allele frequency, %	Database of SNP minor allele frequency in East Asian population, %	*χ*^2^	*P*
*ABCB1* C3435T (rs1045642)		36.1	39.8	0.009	>0.05
CC	189 (40.7)				
CT	215 (46.3)				
TT	60 (12.9)				
*CYP2C19* G681A, CYP2C19*2 (rs4244285)		31.8	31.2	0.693	>0.05
GG	212 (45.7)				
GA	209 (45.0)				
AA	43 (9.3)				
*CYP2C19* G636A, CYP2C19*3 (rs4986893)		3.3	5.6	0.554	>0.05
GG	433 (93.3)				
GA	31 (6.7)				
AA	0				
*CYP2C19* C806T, *CYP2C19* *17 (rs12248560)		0.4	1.5	0.009	>0.05
CC	460 (99.1)				
CT	4 (0.9)				
TT	0				

*Denotes each allele. The rs numbers in parentheses are the accession numbers in the National Center for Biotechnology Information single nucleotide polymorphism (SNP) database.

### Clinical outcomes between the clopidogrel group and ticagrelor group

**Figure 2:**
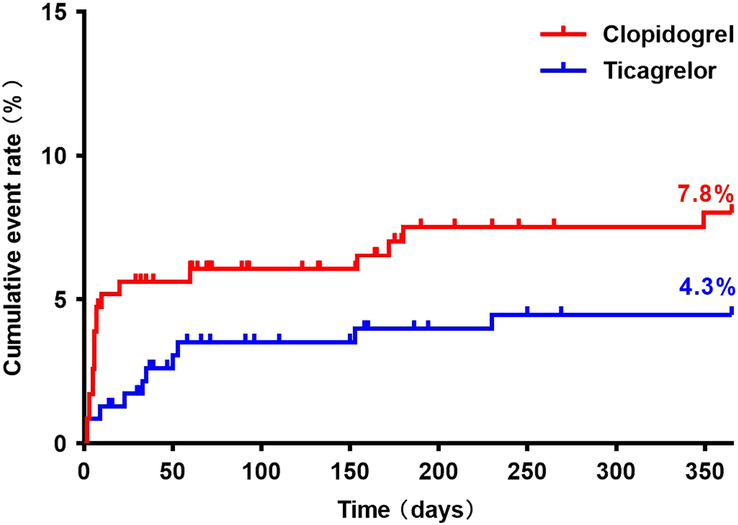
Primary outcome between clopidogrel and ticagrelor groups.

After propensity score matching, a total of 28 composite outcomes of MACE were noted: 18 in the clopidogrel group and 10 in the ticagrelor group. In the clopidogrel group, 12 (5.2%) patients had a stroke, 2 (0.9%) patients presented with a nonfatal myocardial infarction, and 4 (1.7%) patients died from vascular causes. In the ticagrelor group, seven (3.0%) patients had a stroke and three (1.3%) patients died from vascular causes; no patient experienced a nonfatal myocardial infarction. The rate of stroke, nonfatal myocardial infarction, and vascular death after CABG, did not differ between the two treatment groups. The primary outcome, which was a composite of vascular death, myocardial infarction, or stroke, was observed in 7.8% of patients in the clopidogrel group and 4.3% of patients in the ticagrelor group (*P*=0.113) (Fig. 2). For the primary composite endpoint, Kaplan–Meier curves indicated a tendency toward early separation of event rates at 30 days between the clopidogrel and ticagrelor groups [hazard ratio (HR)=2.645, 95% CI: 0.943–7.419, *P*=0.065]. However, the landmark analysis of the primary composite endpoint from day 31 onwards revealed similar event rates at the end of the follow-up between the two groups (HR=0.693, 95% CI: 0.196–2.457, *P*=0.567). Multivariable Cox regression analysis revealed no significant difference between the clopidogrel and ticagrelor groups in 12-month MACE (HR=1.797, 95% CI: 0.826–3.912, *P*=0.139).

### Clinical outcomes in relation to *CYP2C19*

**Figure 3:**
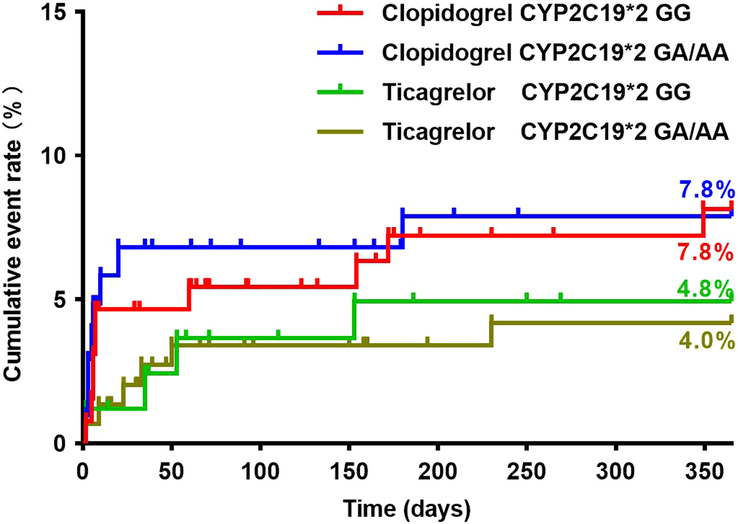
Clinical outcomes in relation to the CYP2C19*2 polymorphisms.

**Figure 4:**
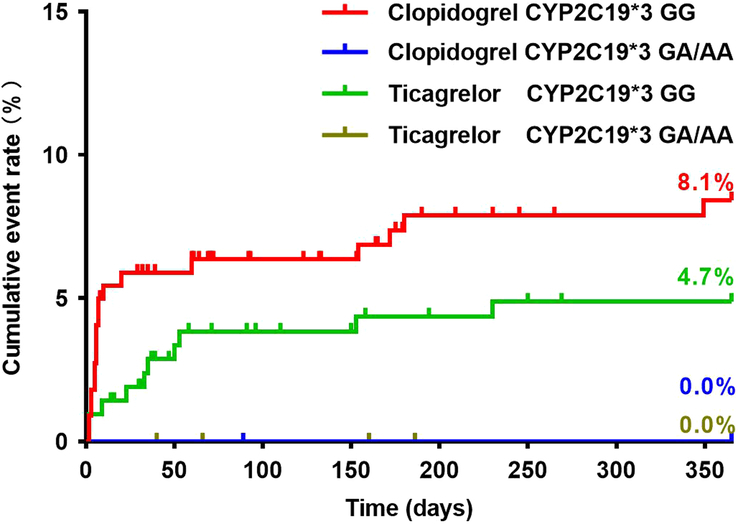
Clinical outcomes in relation to the CYP2C19*3 polymorphisms.

**Figure 5:**
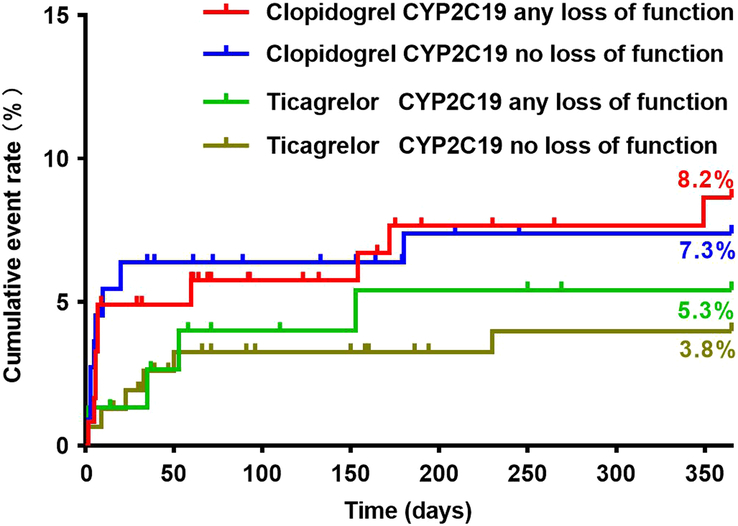
Clinical outcomes in relation to the *CYP2C19* loss of function allele.

**Figure 6:**
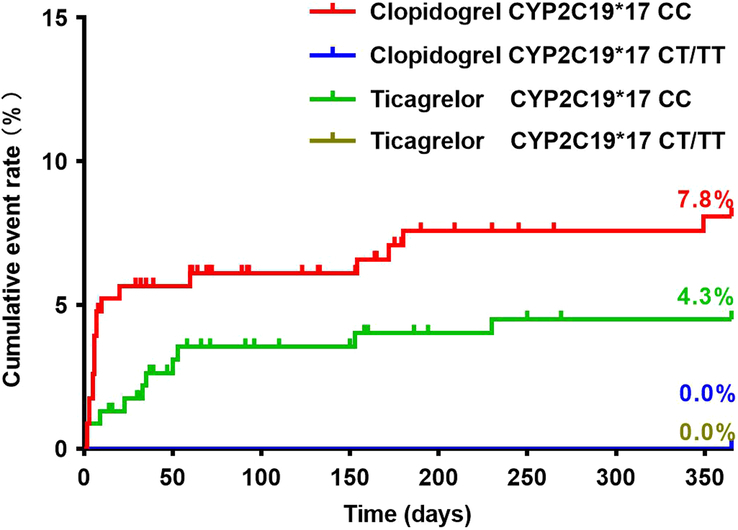
Clinical outcomes in relation to the CYP2C19*17 polymorphisms.

Baseline characteristics of patients did not differ significantly between the clopidogrel and ticagrelor groups considering the *CYP2C19* genotype (see Supplementary Table 1–4, Supplemental Digital Content 2, http://links.lww.com/JS9/C58). There were no significant differences in the incidence of MACE 1-year postsurgery between patients with the CYP2C19*2 GG and those with the CYP2C19*2 GA/AA genotypes, regardless of whether patients had received clopidogrel or ticagrelor (Fig. 3) (Table 3). The number of patients with the CYP2C19*3 GA/AA genotypes was small (clopidogrel: *N*=11; ticagrelor: *N*=20), and none of these patients experienced MACE at 1-year. Patients carrying the CYP2C19*3 GG genotype in the clopidogrel group had a statistically similar incidence of MACE to those carrying the CYP2C19*3 GG genotype in the ticagrelor group (Fig. 4) (Table 3). Patients in the clopidogrel group who had any loss-of-function allele (CYP2C19*2 and CYP2C19*3) had a comparable incidence of 1-year MACE to those in the ticagrelor group (Fig. 5) (Table 3). Cox regression analysis revealed no association between *CYP2C19* *2 or *CYP2C19* *3 genotypes and the incidence of 1-year MACE. Considering the CYP2C19*17 genotype, only four patients carried the CYP2C19*17 CT/TT genotype, with two patients in the clopidogrel group and two in the ticagrelor group (Fig. 6) (Table 3). None of these patients experienced MACE during the 1-year follow-up period. No significant interaction was observed between the *CYP2C19* genotypes and the choice of P2Y_12_ receptor antagonists (*P*>0.05).

**Table 3:** Outcomes in relation to genotypes.

	Aspirin + 75 mg clopidogrel once daily			Aspirin + 90 mg ticagrelor twice daily		
Genotypes	Number of patients	Patients with events	Kaplan–Meier event rate	Number of patients	Patients with events	Kaplan–Meier event rate
Total	232	18 (7.8%)	8.0%	232	10 (4.3%)	4.5%
CYP2C19*2						
GG	129	10 (7.8%)	8.1%	83	4 (4.8%)	4.9%
GA/AA	103	8 (7.8%)	7.9%	149	6 (4.0%)	4.2%
CYP2C19*3						
GG	221	18 (8.1%)	8.4%	212	10 (4.7%)	4.9%
GA/AA	11	0	0	20	0	0
*CYP2C19* loss of function						
Any loss of function	122	10 (8.2%)	8.6%	76	4 (5.3%)	5.4%
No loss of function	110	8 (7.3%)	7.4%	156	6 (3.8%)	4.0%
CYP2C19*17						
CC	230	18 (7.8%)	8.1%	230	10 (4.3%)	4.5%
CT/TT	2	0	0	2	0	0
*ABCB1* C3435T						
CC	99	9 (9.1%)	9.3%	90	8 (8.9)	9.2%
CT/TT	133	9 (6.8%)	7.1%	142	2 (1.4)	1.5%

*Denotes each allele.

### Clinical outcomes in relation to *ABCB1*

**Figure 7:**
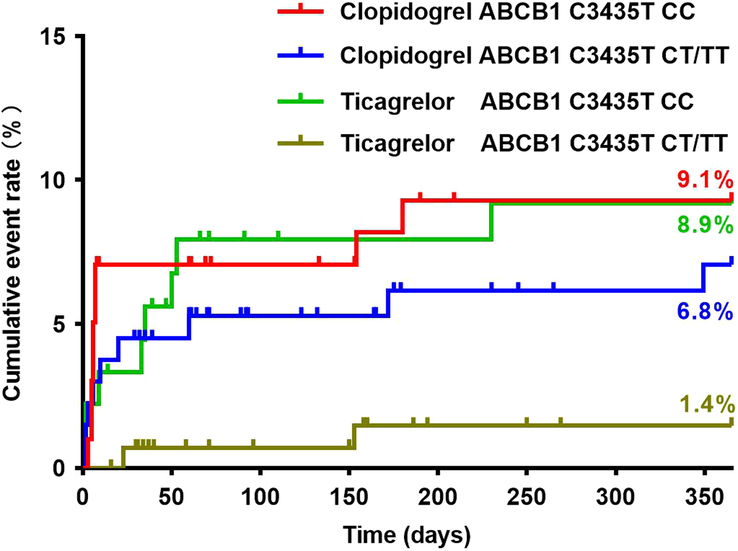
Clinical outcomes in relation to the *ABCB1* 3435 polymorphisms.

Baseline characteristics of patients did not differ significantly between the clopidogrel and ticagrelor groups with respect to *ABCB1* 3435 genotypes (see Supplementary Table 5, Supplemental Digital Content 2, http://links.lww.com/JS9/C58). The rates of MACE were significantly lower in patients carrying the *ABCB1* C3435T CT/TT genotypes in the ticagrelor group than those carrying the *ABCB1* C3435T CC genotype in the clopidogrel group (1.4 vs. 9.1%, adjusted *P*=0.030) and those carrying the *ABCB1* C3435T CC genotype in the ticagrelor group (1.4 vs. 8.9%, adjusted *P*=0.036) (Fig. 7) (Table 3). The Kaplan–Meier event rate was reduced from 9.3% for the *ABCB1* C3435T CC genotype to 7.1% for the *ABCB1* C3435T CT/TT genotypes (HR=0.744, 95% CI: 0.295–1.874, *P* =0.531) in the clopidogrel group, and from 9.2% for the *ABCB1* C3435T CC genotype to 1.5% for the *ABCB1* C3435T CT/TT genotypes (HR=0.151, 95% CI: 0.032–0.713, *P* =0.017) in the ticagrelor group. Based on the Cox regression analysis, the *ABCB1* C3435T CC genotype was significantly associated with the incidence of 1-year MACE (HR=1.558, 95% CI: 1.109–2.188, *P*=0.011). Among patients with *ABCB1* 3435 CC versus CT/TT genotypes, the HR for vascular death was 3.745 (Kaplan–Meier event rates 2.7 *vs.* 0.8%; 95% CI: 0.727–19.231, *P* =0.114), that for myocardial infarction was 1.488 (Kaplan–Meier event rates 0.5 *vs.* 0.4%; 95% CI: 0.093–23.810, *P* =0.773), and that for stroke was 2.045 (Kaplan–Meier event rates 6.2 *vs.* 3.0%; 95% CI: 0.823–5.076, *P* =0.123). Kaplan–Meier curves indicated an early separation of event rates at 30 days between the *ABCB1* 3435 CC and *ABCB1* 3435 CT/TT groups (HR=2.331, 95% CI: 0.903–6.024, *P* =0.080). Similarly, the landmark analysis of the primary composite endpoint from day 31 onwards showed an increased risk of MACE rate for the *ABCB1* 3435 CC group compared with the *ABCB1* 3435 CT/TT group (HR=3.521, 95% CI: 0.910–13.699, *P* =0.068). No significant interaction was detected between the *ABCB1* genotype and the choice of P2Y_12_ receptor antagonists (*P*=0.321).

### Genotypes, antiplatelet therapy, and clinical outcomes

The incidence of 1-year MACE was significantly higher in patients who experienced severe perioperative bleeding than in those without severe perioperative bleeding (14.0 *vs.* 4.9%, adjusted *P*=0.007). The difference was driven by the rise in vascular death and stroke. Rates of myocardial infarction were low in both patients with and without severe perioperative bleeding. The rate of MACE within 30 days or from 30 days to 1-year postsurgery was significantly higher in patients with severe perioperative bleeding than in those without severe perioperative bleeding (within 30 days: 10.5 *vs*. 3.2%, adjusted *P*=0.005; 30 days to 1-year: 8.8 *vs.* 2.0%, adjusted *P*=0.012) (Table 4). Rates of vascular death and stroke were consistently higher in patients with severe perioperative bleeding than in those without severe perioperative bleeding within 30 days or from 30 days to 1-year postsurgery. Univariable and multivariable analyses revealed that the type of antiplatelet therapy (use of clopidogrel and ticagrelor) was not associated with the incidence of MACE or perioperative bleeding. Instead, age >65 years [odds ratio (OR)=3.090, 95% CI: 1.141–8.371, *P*=0.027), stroke history (OR=2.382, 95% CI: 0.941–6.028, *P*=0.067), and the *ABCB1* C3435T genotype (CT/TT *vs.* CC: OR=0.388, 95% CI: 0.176–0.859, *P*=0.020) were related to the risk of MACE (Table 5). Male sex (OR=0.397, 95% CI: 0.220–0.715, *P*=0.002) and the *ABCB1* C3435T genotype (CT/TT *vs.* CC: OR=0.568, 95% CI: 0.323–0.998, *P*=0.049) were related to severe perioperative bleeding (Table 6).

**Table 4:** Bleeding and outcomes.

Outcomes	No UDPB-defined 3 or 4 bleeding (*N*=407)	UDPB-defined 3 or 4 bleeding (*N*=57)	*P*	Adjusted *P* ^a^
1-year outcome				
Vascular death, stroke, or myocardial infarction	20 (4.9)	8 (14.0)	0.014	0.007
Vascular death	4 (1.0)	3 (5.3)	0.043	0.043
Stroke	14 (3.4)	5 (8.8)	0.070	0.049
Myocardial infarction	2 (0.5)	0	1.000	0.997
30-day outcome				
Vascular death, stroke, or myocardial infarction	13 (3.2)	6 (10.5)	0.020	0.005
Vascular death	2 (0.5)	1 (1.8)	0.326	0.393
Stroke	10 (2.5)	5 (8.8)	0.027	0.004
Myocardial infarction	1 (0.2)	0	1.000	0.997
30 days to 1 year outcome				
Vascular death, stroke, or myocardial infarction	8 (2.0)	5 (8.8)	0.014	0.012
Vascular death	2 (0.5)	2 (3.5)	0.076	0.056
Stroke	5 (1.2)	3 (5.3)	0.063	0.066
Myocardial infarction	1 (0.2)	0	1.000	1.000

UDPB, universal definition of perioperative bleeding.

a Adjustment with age >65, sex, hypertension, diabetes mellitus, acute coronary syndrome and use of proton pump inhibitor.

**Table 5:** Univariable and multivariable analyses to MACE.

	Univariable analysis			Multivariable analysis		
	OR	95% CI	*P*	OR	95% CI	*P*
Age >65 years	3.174	1.184–8.505	0.016	3.090	1.141–8.371	0.027
Male	1.786	0.605–5.268	0.356			
Hypertension	1.016	0.436–2.367	1.000			
Diabetes mellitus	1.448	0.669–3.133	0.436			
Hyperlipidemia	0.885	0.391–2.004	0.840			
Stroke history	2.516	1.019–6.213	0.068	2.382	0.941–6.028	0.067
Acute coronary syndrome	1.494	0.694–3.215	0.331			
Percutaneous coronary intervention history	0.617	0.181–2.098	0.597			
Number of grafts	0.870	0.549–1.379	0.552			
Use of artery graft	1.067	1.041–1.092	0.614			
Proton pump inhibitor	3.031	0.402–22.848	0.504			
Use of clopidogrel (vs. ticagrelor)	1.867	0.843–4.137	0.171			
Statin use	1.642	0.214–12.585	1.000			
*CYP2C19* loss-of-function (CYP2C19*2 or *3	0.730	0.340–1.569	0.436			
*ABCB1* C3435T CT/TT (vs. CC)	0.422	0.193–0.922	0.030	0.388	0.176–0.859	0.020

*Denotes each allele.

**Table 6:** Univariable and multivariable analyses to UDPB-defined class 3 or class 4 perioperative bleeding.

	Univariable analysis			Multivariable analysis		
	OR	95% CI	*P*	OR	95% CI	*P*
Age >65 years	1.477	0.817–2.672	0.247			
Male	0.401	0.223–0.721	0.003	0.397	0.220–0.715	0.002
Hypertension	0.949	0.518–1.741	0.877			
Diabetes mellitus	1.029	0.591–1.792	1.000			
Hyperlipidemia	0.576	0.305–1.088	0.102			
Stroke history	1.166	0.522–2.604	0.671			
Acute coronary syndrome	1.597	0.915–2.787	0.117			
Percutaneous coronary intervention history	0.587	0.242–1.421	0.333			
Number of grafts	1.033	0.735–1.452	0.853			
Use of artery graft	2.413	0.278–16.538	0.706			
Proton pump inhibitor	1.134	0.428–3.002	0.822			
Use of clopidogrel (vs. ticagrelor)	1.560	0.888–2.742	0.157			
Statin use	3.665	0.487–27.582	0.231			
*CYP2C19* loss-of-function (CYP2C19*2 or *3)	1.041	0.790–2.457	0.260			
*ABCB1* C3435T CT/TT (vs. CC)	0.577	0.331–1.007	0.061	0.568	0.323-0.998	0.049

*Denotes each allele.

## Discussion

In recent years, DAPT has become the standard therapy and guideline recommendation following both ACS and/or PCI^24,25^. However, the optimal antiplatelet strategy post-CABG remains controversial. Despite conflicting results regarding DAPT versus single antiplatelet therapy post-CABG, it is recommended that P2Y_12_ receptor inhibitor therapy should be resumed postsurgery when deemed safe and continued for up to 12 months in patients with ACS undergoing CABG^11,14^. In addition, for patients undergoing OPCABG, dual antiplatelet therapy with a combination of aspirin and clopidogrel is recommended for one-year period^12^. With a faster onset of action and more pronounced platelet inhibition than clopidogrel, ticagrelor has been extensively utilized in patients with ACS or PCI in combination with aspirin. Within the ACS setting, ticagrelor was found to be associated with lower MACE and even lower all-cause mortality than clopidogrel^15,26^. However, there is still a lack of clinical studies comparing differences between clopidogrel plus aspirin and ticagrelor plus aspirin after CABG.

In the present study, the clopidogrel group had a higher incidence of MACE than the ticagrelor group; however, there were no significant differences between the two groups (7.8 *vs.* 4.3%, *P*=0.113). This finding supports the results of several recent studies. Chang *et al*.^27^ have demonstrated that there were no significant differences in MACE or major bleeding at 1-year after CABG in 259 patients treated with DAPT comprising aspirin and clopidogrel or aspirin and ticagrelor (HR=0.67, 95% CI: 0.24–1.87, *P*=0.442). Another trial enrolled 147 consecutive patients who were randomised into the ticagrelor group (receiving aspirin 100 mg/day plus ticagrelor 2×90 mg/day) or the clopidogrel group (receiving aspirin 100 mg/day plus clopidogrel 75 mg/day). The authors found that the incidence of MACE did not differ significantly between the two groups (ticagrelor group *vs*. clopidogrel group: 2.9 *vs*. 1.3%, *P*=0.505) 1-year post-CABG^28^. Furthermore, a previous analysis of the Platelet Inhibition and Patient Outcomes (PLATO) trial showed that 13.1% of patients who received clopidogrel and aspirin and 10.6% of patients who received ticagrelor and aspirin experienced the primary objective of cardiovascular death, myocardial infarction, or stroke (HR=0.84, 95% CI: 0.60–1.16, *P*=0.286). The PLATO trial analysis indicated that cardiovascular death decreased from 7.9% for clopidogrel to 4.1% for ticagrelor (HR=0.52; 95% CI: 0.32–0.85, *P*<0.01), and total mortality decreased from 9.7 to 4.7% (HR=0.49; 95% CI: 0.32–0.77, *P*<0.01)^3^. In the present study, we did not find a significant difference in vascular death between the clopidogrel and ticagrelor groups, which may be due to the low incidence of mortality (clopidogrel *vs.* ticagrelor: 1.7 *vs.* 1.3%). The incidence of MACE and all-cause mortality (including cardiovascular and noncardiovascular death) in our study was lower than that reported in the analysis of the PLATO trial^3^, which could be attributed to several reasons. For example, the Asian population in our study may have a lower rate of MACE post-CABG than other populations^27,29^. Furthermore, our study included non-ACS patients, which could be responsible for the lower incidence of postoperative MACE and all-cause mortality. During the first 30 days of treatment with antiplatelet therapy after surgery, although there was no statistically significant difference, a numerically higher rate of MACE was recorded in the clopidogrel group than in the ticagrelor group. However, when the full follow-up period was considered, the difference in outcomes between the clopidogrel and ticagrelor groups was not significant. CABG surgery is often considered a high-risk procedure, associated with a 30-day morbidity rate of up to 14.0% and a mortality rate of up to 2.0%^30^. The potent anti-inflammatory and antiplatelet effects of ticagrelor may offer greater benefits in reducing cardiovascular adverse events within the initial 30 days after surgery^31^. However, during a longer-term antiplatelet treatment, patients who underwent CABG tended to have more stable hemodynamics^32^. This stability could reduce the disparities among various P2Y_12_ receptor antagonists, resulting in no significant difference in the 1-year prognosis between the clopidogrel and ticagrelor treatments.

In patients with coronary artery disease, the findings of previous clinical studies investigating the selection of a P2Y_12_ receptor antagonist in combination with aspirin remain controversial. This discrepancy could be attributed to variations in interindividual responses to P2Y_12_ receptor antagonists^33,34^. Compared with ticagrelor, which usually does not require metabolic activation, clopidogrel is an orally administered prodrug that needs to undergo metabolism before inhibiting platelet aggregation. Variations in the genes regulating clopidogrel absorption (*ABCB1*) and metabolic activation (*CYP2C19*) have been implicated in the modulation of clopidogrel absorption and metabolism and are associated with clinical outcomes^35–38^. Currently, there is still a lack of studies investigating the impact of these genetic variations on clinical outcomes in patients who have undergone CABG surgery. Our findings suggest that variants in *CYP2C19* (*2, *3, and *17) did not influence the 1-year clinical outcomes in patients post-OPCABG, regardless of the P2Y_12_ receptor antagonist employed. Previous research has shown that patients with an acute myocardial infarction who were receiving clopidogrel and carrying *CYP2C19* loss-of-function alleles (*2 or *3) had a higher rate of subsequent cardiovascular events than those without these alleles. This effect was particularly pronounced among patients undergoing PCI^9^. However, during a 1-year antiplatelet treatment, *CYP2C19* seems to have a moderate effect on platelet aggregation^39^. Additionally, for patients with multivessel coronary disease, OPCABG appeared to provide satisfactory long-term outcomes that are superior to those of PCI. This might explain the restricted effect of *CYP2C19* polymorphisms on clinical events in our study^40^. Nevertheless, patients carrying the *ABCB1* C3435T CC genotype had a higher incidence of MACE than those carrying the *ABCB1* C3435T CT/TT genotype. This trend was consistent for each endpoint, both within 30 days and from 30 days to 1-year postsurgery. These results are contrary to those reported previously. According to several previous studies, patients with the *ABCB1* C3435T TT genotype who were treated with clopidogrel for ACS responded poorly to clopidogrel and had an increased risk of MACE^41,42^. The discrepancies between the findings of previous studies and those of our study could be attributed to the distinct prognosis of patient who had undergone cardiac surgery and those without cardiac surgery. Among patients who have undergone cardiac surgery, perioperative complications, particularly bleeding, can crucially impact the prognosis of patients undergoing cardiac surgery. This was identified in our study, revealing that patients who experienced severe perioperative bleeding were more likely to experience MACE during the follow-up period. Perioperative bleeding and the use of blood products after cardiac surgery are recognized as important determinants of outcomes after cardiac interventions^43^. According to a recent study including 3988 patients who underwent OPCABG, severe perioperative bleeding was associated with an increased risk of in-hospital death and postoperative myocardial infarction^44^. Perioperative bleeding can be influenced by various factors, including but not limited to antiplatelet therapy (clopidogrel and ticagrelor), surgical techniques (on-pump and off-pump procedures), patient comorbidities (ACS and critical preoperative state), and other medications (low-molecular-weight heparin, fondaparinux, and unfractionated heparin use)^45,46^. For example, in a previous study, researchers found that the overall risk of major CABG-related bleeding complications was lower with ticagrelor than with clopidogrel and the difference was driven by a lower incidence with ticagrelor than with clopidogrel when the platelet inhibitor was discontinued 72–120 h before surgery^47^. However, our previous study showed that in the setting of OPCABG, there were no significant differences in severe perioperative bleeding between clopidogrel and ticagrelor groups^48^. Based on the findings of the current study, the multivariable analysis showed that the genetic factor, that is, the *ABCB1* 3435 CC genotype, may be another potential factor related to perioperative bleeding, consistent with the findings in our previous analysis^49^. *ABCB1* encodes the P-glycoprotein efflux transporter, and genetic polymorphisms in *ABCB1* can affect P-gp activity^50^. The C3435T variant in *ABCB1* is one of the most studied polymorphisms in pharmacogenetic research, and has been associated with altered disposition of several drugs, including clopidogrel and ticagrelor^51,52^. Among clopidogrel-treated patients, patients with the *ABCB1* 3435 TT genotype exhibited lower maximum concentration (Cmax) and area under the curve (AUC) values of clopidogrel and its active metabolite than carriers of the *ABCB1* 3435 CT/CC genotype^32^. This observation could explain the high-risk of bleeding in patients with the *ABCB1* 3435 CC/CT genotype^53^. However, given the lack of preoperative data for included patients (like the type of preoperative antiplatelet therapy and cessation time of antiplatelet drugs) and to ensure the accuracy of the analysis, we did not perform further analysis of these factors that impact perioperative bleeding in the current study.

Herein, the *ABCB1* 3435 CC genotype appeared to increase the risk of both bleeding and MACE events. This may seem contradictory; however, bleeding events in the current study referred to perioperative bleeding events, and the sequence and timing of bleeding events and MACE events may provide some explanation. We defined the perioperative bleeding events as those that occurred during the perioperative period, that is, the amount of blood loss and blood transfusion, typically occurring within 24 h postsurgery, rather than the bleeding events that were followed up within 1-year postsurgery. The *ABCB1* 3435 CC genotype was related to these perioperative bleeding events, and the perioperative bleeding complications, including re-exploration due to bleeding, the perioperative use of blood products, and bleeding from vital organs, can increase the risk of poor outcomes and the incidence of MACE in patients undergoing cardiac surgery^54–56^. Therefore, *ABCB1* 3435 CC may exert an indirect role in terms of increasing the incidence of MACE events.

Our study had several limitations. First, this was a single-center, retrospective study with a small sample size. Although propensity score matching analysis was used to reduce the bias, it was not possible to eliminate unknown confounding factors. Second, due to the retrospective nature of the study, we were unable to determine which P2Y_12_ receptor antagonist (clopidogrel or ticagrelor) was administered prior to OPCABG surgery, and the precise duration of days for patients to discontinue antiplatelet drugs before surgery was unclear, even though the standard procedure was to discontinue ticagrelor for at least 3 days and discontinue clopidogrel for at least 5 days before surgery. Third, we were unable to perform a more detailed analysis of the cause of perioperative bleeding given the lack of preoperative data of patients in the current study (like types of preoperative antiplatelet therapy and cessation times of antiplatelet drugs). Additionally, our study only included patients who underwent OPCABG procedures, as this is the predominant method of coronary bypass grafting performed at our center. Considering that the prognosis between on-pump and off-pump procedures remains controversial, more studies are needed in the future. Another limitation was that because of the absence of 1-year coronary computed tomography angiography data for many patients, we did not know the patency of the grafts and could not further explore the influence of different P2Y_12_ receptor antagonists on vascular conditions.

## Conclusion

In the current study, there were no significant differences in 1-year MACE between patients receiving aspirin plus clopidogrel and those receiving aspirin plus ticagrelor after OPCABG. Patients with the *ABCB1* CC genotype had a higher risk of MACE than those carrying the *ABCB1* CT/TT genotype. The impact of genotypes on the postoperative prognosis of CABG still warrants further investigation.

## Ethical approval

The protocol was approved by Ethics Committee of Zhongshan Hospital, Fudan University (B2021-453).

## Consent

Written informed consent was obtained from the patient for publication of this case report and accompanying images. A copy of the written consent is available for review by the Editor-in-Chief of this journal on request.

## Sources of funding

This study was supported by the Project of Shanghai Municipal health planning commission (NO.2016ZB0301); Project of Clinical Key Subject Construction of Shanghai (shslczdzk06504).

## Author contribution

Z.W. and R.M.: study design, data collection, data analysis and interpretation, writing the paper; X.L.: data collection, data analysis, and writing the paper; X.L. and Q.X.: data collection, data interpretation, and writing the paper; Y.Y.: data collection, data analysis, and writing the paper; C.W. and Q.L.: study conception and design, data collection, data analysis and interpretation, and writing the paper.

## Conflicts of interest disclosure

There are no conflicts of interest.

## Research registration unique identifying number (UIN)

ChiCTR2100048609.

## Guarantor

Qianzhou Lv, Zhongshan hospital, Fudan University. E-mail:lv.qianzhou@zs-hospital.sh.cn.

## Data availability statement

Data are held by the study sponsor and are available upon reasonable request.

## Provenance and peer review

Not commissioned, externally peer-reviewed.

## Assistance with the study

None.

## Supplementary Material
