# Supplementary material for: Clinical efficacy of clopidogrel and ticagrelor in patients undergoing off-pump coronary artery bypass grafting: a retrospective cohort study
Source: Int J Surg. 2024 Mar 4;110(6):3450–60. doi: 10.1097/JS9.0000000000001246 (PMC11175730; doi:10.1097/JS9.0000000000001246)
Supplement: Supplementary file 2 [file js9-110-3450-s002.docx]

**Supplementary tables**

**Supplementary Table 1 Baseline characteristics of patients in relation to *CYP2C19**2**

| **Variables** | **Clopidogrel *CYP2C19**2 GG (N = 129)** | **Clopidogrel *CYP2C19**2 GA/AA (N = 103)** | **Ticagrelor *CYP2C19**2 GG (N = 83)** | **Ticagrelor *CYP2C19**2 GA/AA (N = 149)** | **P** |
| --- | --- | --- | --- | --- | --- |
| **Age >65 yrs** | 83 (64.3) | 65 (63.1) | 49 (59.0) | 84 (56.4) | 0.529 |
| **Male, n (%)** | 105 (81.4) | 81 (78.6) | 65 (78.3) | 109 (73.2) | 0.417 |
| **BMI, kg/m^2^** | 24.60 ± 3.02 | 24.07 ± 2.80 | 24.77 ± 3.70 | 24.46 ± 2.95 | 0.432 |
| **Hypertension** | 92 (71.3) | 76 (73.8) | 53 (63.9) | 109 (73.2) | 0.427 |
| **DM** | 66 (51.2) | 45 (43.7) | 38 (45.8) | 76 (51.0) | 0.587 |
| **Hyperlipidemia** | 37 (28.7) | 38 (36.9) | 26 (31.3) | 60 (40.3) | 0.192 |
| **Stroke history** | 19 (14.7) | 12 (11.7) | 7 (8.4) | 20 (13.4) | 0.568 |
| **ACS** | 49 (38.0) | 43 (41.7) | 45 (54.2) | 68 (45.6) | 0.123 |
| **PCI history** | 17 (13.2) | 19 (18.4) | 9 (10.8) | 29 (19.5) | 0.241 |
| **Number of grafts** | 2.82 ± 0.77 | 3.07 ± 0.80 | 3.14 ± 0.96 | 2.86 ± 0.76 | 0.008* |
| **HB** | 130.95 ± 16.31 | 131.86 ± 15.61 | 130.76 ± 13.00 | 129.77 ± 14.18 | 0.744 |
| **RBC** | 4.29 ± 0.54 | 4.33 ± 0.54 | 4.34 ± 0.48 | 4.28 ± 0.45 | 0.741 |
| **APTT** | 27.21 ± 2.26 | 27.00 ± 2.77 | 27.06 ± 2.46 | 26.97 ± 2.33 | 0.857 |
| **PT** | 11.90 ± 0.82 | 11.72 ± 0.99 | 11.71 ± 0.88 | 11.76 ± 0.87 | 0.311 |
| **PPI** | 114 (88.4) | 91 (88.3) | 77 (92.8) | 137 (91.9) | 0.570 |
| **Statin use** | 119 (92.2) | 98 (95.1) | 78 (94.0) | 143 (96.0) | 0.587 |

BMI: body mass index; DM: diabetes mellitus; ACS: acute coronary syndrome; PCI: percutaneous coronary intervention; HB: hemoglobin; RBC: red blood cell; APTT: activated partial thromboplastin time; PT: prothrombin time; PPI: proton pump inhibitor.

*Post hoc pairwise tests with Bonferroni correction indicated that there was no significant difference between the two groups (adjusted P>0.05)

**Supplementary Table 2 Baseline characteristics of patients in relation to *CYP2C19**3**

| **Variables** | **Clopidogrel *CYP2C19**3 GG (N = 221)** | **Clopidogrel *CYP2C19**3 GA/AA (N = 11)** | **Ticagrelor *CYP2C19**3 GG (N = 212)** | **Ticagrelor *CYP2C19**3 GA/AA (N = 20)** | **P** |
| --- | --- | --- | --- | --- | --- |
| **Age >65 yrs** | 140 (63.3) | 8 (72.7) | 121 (57.1) | 12 (60.0) | 0.495 |
| **Male, n (%)** | 176 (79.6) | 10 (90.9) | 159 (75.0) | 15 (75.0) | 0.494 |
| **BMI, kg/m^2^** | 24.36 ± 2.98 | 24.46 ± 1.90 | 24.55 ± 3.06 | 24.72 ± 4.82 | 0.904 |
| **Hypertension** | 158 (71.5) | 10 (90.9) | 150 (70.8) | 12 (60.0) | 0.355 |
| **DM** | 105 (47.5) | 6 (54.5) | 104 (49.1) | 10 (50.0) | 0.958 |
| **Hyperlipidemia** | 72 (32.6) | 3 (27.3) | 78 (36.8) | 8 (40.0) | 0.717 |
| **Stroke history** | 27 (12.2) | 4 (36.4) | 25 (11.8) | 2 (10.0) | 0.155 |
| **ACS** | 88 (39.8) | 4 (36.4) | 104 (49.1) | 9 (45.0) | 0.252 |
| **PCI history** | 35 (15.8) | 1 (9.1) | 32 (15.1) | 6 (30.0) | 0.357 |
| **Number of grafts** | 2.93 ± 0.79 | 3.00 ± 0.78 | 2.98 ± 0.85 | 2.80 ± 0.83 | 0.780 |
| **HB** | 130.95 ± 16.20 | 139.63 ± 6.61 | 129.52 ± 13.77 | 136.55 ± 11.98 | 0.037* |
| **RBC** | 4.30 ± 0.55 | 4.56 ± 0.27 | 4.29 ± 0.45 | 4.50 ± 0.50 | 0.098 |
| **APTT** | 27.08 ± 2.46 | 27.92 ± 2.06 | 26.96 ± 2.37 | 27.41 ± 2.43 | 0.535 |
| **PT** | 11.83 ± 0.91 | 11.64 ± 0.73 | 11.74 ± 0.89 | 11.80 ± 0.71 | 0.685 |
| **PPI** | 195 (88.2) | 10 (90.9) | 196 (92.5) | 18 (90.0) | 0.456 |
| **Statin use** | 207 (93.7) | 10 (90.9) | 201 (94.8) | 20 (100.0) | 0.566 |

BMI: body mass index; DM: diabetes mellitus; ACS: acute coronary syndrome; PCI: percutaneous coronary intervention; HB: hemoglobin; RBC: red blood cell; APTT: activated partial thromboplastin time; PT: prothrombin time; PPI: proton pump inhibitor.

*Post hoc pairwise tests with Bonferroni correction indicated that there was no significant difference between the two groups (adjusted P>0.05)

**Supplementary Table 3 Baseline characteristics of patients in relation to *CYP2C19* loss-of-function alleles**

| **Variables** | **Clopidogrel *CYP2C19* any loss of function (N = 122)** | **Clopidogrel *CYP2C19* no loss of function (N = 110)** | **Ticagrelor *CYP2C19* any loss of function (N = 76)** | **Ticagrelor *CYP2C19* no loss of function (N = 156)** | **P** |
| --- | --- | --- | --- | --- | --- |
| **Age >65 yrs** | 78 (63.9) | 70 (63.6) | 44 (57.9) | 89 (57.1) | 0.564 |
| **Male, n (%)** | 99 (81.1) | 87 (79.1) | 61 (80.3) | 113 (72.4) | 0.294 |
| **BMI, kg/m^2^** | 24.59 ± 3.08 | 24.11 ± 2.74 | 24.77 ± 3.42 | 24.47 ± 3.14 | 0.496 |
| **Hypertension** | 86 (70.5) | 82 (74.5) | 49 (64.5) | 113 (72.4) | 0.487 |
| **DM** | 62 (50.8) | 49 (44.5) | 34 (44.7) | 80 (51.3) | 0.604 |
| **Hyperlipidemia** | 35 (28.7) | 40 (36.4) | 23 (30.3) | 63 (40.4) | 0.176 |
| **Stroke history** | 17 (13.9) | 14 (12.7) | 6 (7.9) | 21 (13.5) | 0.620 |
| **ACS** | 47 (38.5) | 45 (40.9) | 42 (55.3) | 71 (45.5) | 0.114 |
| **PCI history** | 16 (13.1) | 20 (18.2) | 7 (9.2) | 31 (19.9) | 0.137 |
| **Number of grafts** | 2.84 ± 0.78 | 3.04 ± 0.79 | 3.20 ± 0.95 | 2.85 ± 0.77 | 0.004 |
| **HB** | 130.61 ± 16.67 | 132.19 ± 15.20 | 130.31 ± 13.02 | 130.03 ± 14.13 | 0.691 |
| **RBC** | 4.28 ± 0.56 | 4.34 ± 0.53 | 4.33 ± 0.48 | 4.29 ± 0.45 | 0.776 |
| **APTT** | 27.21 ± 2.17 | 27.02 ± 2.72 | 27.05 ± 2.51 | 26.98 ± 2.31 | 0.884 |
| **PT** | 11.91 ± 0.82 | 11.72 ± 0.98 | 11.70 ± 0.91 | 11.76 ± 0.86 | 0.292 |
| **PPI** | 108 (88.5) | 97 (88.2) | 71 (93.4) | 143 (91.7) | 0.540 |
| **Statin use** | 113 (92.6) | 104 (94.5) | 71 (93.4) | 150 (96.2) | 0.585 |

BMI: body mass index; DM: diabetes mellitus; ACS: acute coronary syndrome; PCI: percutaneous coronary intervention; HB: hemoglobin; RBC: red blood cell; APTT: activated partial thromboplastin time; PT: prothrombin time; PPI: proton pump inhibitor.

*Post hoc pairwise tests with Bonferroni correction indicated that there was no significant difference between the two groups (adjusted P>0.05)

**Supplementary Table 4 Baseline characteristics of patients in relation to *CYP2C19**17**

| **Variables** | **Clopidogrel *CYP2C19**17 CC (N = 230)** | **Clopidogrel *CYP2C19**17 CT/TT (N = 2)** | **Ticagrelor *CYP2C19**17 CC (N = 230)** | **Ticagrelor *CYP2C19**17 CT/TT (N = 2)** | **P** |
| --- | --- | --- | --- | --- | --- |
| **Age >65 yrs** | 146 (63.5) | 2 (100.0) | 131 (57.0) | 2 (100.0) | 0.227 |
| **Male, n (%)** | 184 (80.0) | 2 (100.0) | 172 (74.8) | 2 (100.0) | 0.454 |
| **BMI, kg/m^2^** | 24.38 ± 2.94 | / | 24.60 ± 3.21 | / | / |
| **Hypertension** | 167 (72.6) | 1 (50.0) | 161 (70.0) | 1 (50.0) | 0.499 |
| **DM** | 109 (47.4) | 2 (100.0) | 114 (49.6) | 0 | 0.302 |
| **Hyperlipidemia** | 75 (32.6) | 0 | 86 (37.4) | 0 | 0.443 |
| **Stroke history** | 31 (13.5) | 0 | 26 (11.3) | 1 (50.0) | 0.310 |
| **ACS** | 92 (40.0) | 0 | 113 (49.1) | 0 | 0.044 |
| **PCI history** | 36 (15.7) | 0 | 38 (16.5) | 0 | 0.950 |
| **Number of grafts** | 2.93 ± 0.79 | / | 2.97 ± 0.85 | / | / |
| **HB** | 131.28 ± 16.03 | / | 129.94 ± 13.66 | / | / |
| **RBC** | 4.31 ± 0.55 | / | 4.30 ± 0.45 | / | / |
| **APTT** | 27.12 ± 2.45 | / | 26.99 ± 2.38 | / | / |
| **PT** | 11.82 ± 0.90 | / | 11.74 ± 0.88 | / | / |
| **PPI** | 204 (88.7) | 1 (50.0) | 212 (92.2) | 2 (100.0) | 0.143 |
| **Statin use** | 215 (93.5) | 2 (100.0) | 219 (95.2) | 2 (100.0) | 0.639 |

BMI: body mass index; DM: diabetes mellitus; ACS: acute coronary syndrome; PCI: percutaneous coronary intervention; HB: hemoglobin; RBC: red blood cell; APTT: activated partial thromboplastin time; PT: prothrombin time; PPI: proton pump inhibitor.

**Supplementary Table 5 Baseline characteristics of patients in relation to *ABCB1* C3435T**

| **Variables** | **Clopidogrel *ABCB1* C3435T CC (N = 99)** | **Clopidogrel *ABCB1* C3435T CT/TT (N = 133)** | **Ticagrelor *ABCB1* C3435T CC (N = 90)** | **Ticagrelor** ***ABCB1* C3435T CT/TT (N =142)** | **P** |
| --- | --- | --- | --- | --- | --- |
| **Age >65 yrs** | 62 (62.6) | 48 (53.3) | 86 (64.7) | 85 (59.9) | 0.375 |
| **Male, n (%)** | 78 (78.8) | 69 (76.7) | 108 (81.2) | 105 (73.9) | 0.532 |
| **BMI, kg/m^2^** | 24.41 ± 2.95 | 24.34 ± 2.93 | 24.20 ± 3.16 | 24.80 ± 3.26 | 0.434 |
| **Hypertension** | 68 (68.7) | 59 (65.6) | 100 (75.2) | 103 (72.5) | 0.413 |
| **DM** | 50 (50.5) | 49 (54.4) | 61 (45.9) | 65 (45.8) | 0.527 |
| **Hyperlipidemia** | 35 (35.4) | 34 (37.8) | 40 (30.1) | 52 (36.6) | 0.597 |
| **Stroke history** | 11 (11.1) | 9 (10.0) | 20 (15.0) | 18 (12.7) | 0.688 |
| **ACS** | 45 (45.5) | 45 (50.0) | 47 (35.3) | 68 (47.9) | 0.098 |
| **PCI history** | 15 (15.2) | 15 (16.7) | 21 (15.8) | 23 (16.2) | 0.994 |
| **Number of grafts** | 2.87 ± 0.83 | 2.98 ± 0.75 | 2.83 ± 0.92 | 3.04 ± 0.80 | 0.191 |
| **HB** | 131.91 ± 16.18 | 130.95 ± 15.87 | 127.77 ± 14.97 | 131.62 ± 12.75 | 0.193 |
| **RBC** | 4.33 ± 0.57 | 4.30 ± 0.53 | 4.26 ± 0.50 | 4.33 ± 0.43 | 0.770 |
| **APTT** | 26.85 ± 2.30 | 27.31 ± 2.54 | 26.77 ± 2.56 | 27.15 ± 2.24 | 0.294 |
| **PT** | 11.78 ± 1.05 | 11.85 ± 0.78 | 11.71 ± 1.00 | 11.76 ± 0.78 | 0.677 |
| **PPI** | 83 (83.9) | 86 (95.6) | 122 (91.7) | 128 (90.1) | 0.054 |
| **Statin use** | 91 (91.9) | 85 (94.4) | 126 (94.7) | 136 (95.8) | 0.648 |

BMI: body mass index; DM: diabetes mellitus; ACS: acute coronary syndrome; PCI: percutaneous coronary intervention; HB: hemoglobin; RBC: red blood cell; APTT: activated partial thromboplastin time; PT: prothrombin time; PPI: proton pump inhibitor.
